# Species associated with whale sharks *Rhincodontypus* (Orectolobiformes, Rhincodontidae) in the Galapagos Archipelago

**DOI:** 10.3897/BDJ.11.e97864

**Published:** 2023-03-08

**Authors:** Sofia M Green, Alex Hearn, Jonathan R Green

**Affiliations:** 1 Galapagos Whale Shark Project, Puerto Ayora, Ecuador Galapagos Whale Shark Project Puerto Ayora Ecuador; 2 Universidad San Francisco de Quito, Quito, Ecuador Universidad San Francisco de Quito Quito Ecuador

**Keywords:** interspecific interactions, natural history, parasitism, species associations, symbiosis

## Abstract

Whale sharks *Rhincodontypus* frequently appear to interact or associate with other species, which vary depending on the community structure and the demographic of the whale sharks at each location globally. Here, we present the species sighted frequently around whale sharks in the Galapagos Archipelago and reported by dive guides and scientists and also in earlier publications. These associated species include cetacean species: bottlenose dolphins *Tursiopstruncatus*, other shark species: silky sharks *Carcharhinusfalciformis*, Galapagos sharks *Carcharhinusgalapagensis*, scalloped hammerhead sharks *Sphyrnalewini*, tiger sharks *Galeocerdocuvier* and teleost fish species: remoras *Remora* remora, yellowfin tuna *Thunnusalbacares*, almaco jacks *Seriolarivoliana* and black jacks *Caranxlugubris*. The recording of interspecies associations and interactions may lead to better understanding of the natural history of whale sharks and can show important symbiotic relationships or interdependence between different species.

## Introduction

The whale shark *Rhincodontypus* Smith 1828, is a circumglobal tropical and temperate water species (Rowat and Brooks 2012). It is mostly solitary and pelagic, although it also forms seasonal feeding aggregations of mostly immature males in at least a dozen (mostly coastal) sites (e.g. Belize, Heyman et al. (2001); Gulf of Mexico, Hoffmayer et al. (2007); Tanzania, Rohner et al. (2015)). Several species are known to associate with whale sharks around the globe, yet they vary across different locations. Bottlenose dolphins *Tursiopstruncatus* (Montagu, 1821) have been reported swimming with whale sharks or feeding alongside them in the Gulf of Mexico, Belize, Honduras, the Red Sea and Christmas Island (Latusek-Nabholz 2014). Cleaning interactions have been reported by Araujo et al. (2020) of the blue-streak cleaner wrasse *Labroidesdimidiatus* (Valenciennes, 1839) and the moon wrasse *Thalassomalunare* (Linnaeus, 1758) in the Philippines and by Quimbayo et al. (2016) of the King Angelfish *Holacanthuspasser* Valenciennes, 1846 in Malpelo Island. Other associations recorded include suckerfish *Remora* spp. and *Echeneisnaucrates* Linnaeus, 1758 in north-western Australia (Norman 1999), schooling fish such as tuna and mackerels, plus carcharhinid shark species swimming alongside whale sharks off south Texas (Hoffman et al. 1981) and more tuna species associations in the Atlantic and Indian Oceans (Escalle et al. 2018). Indeed, until recently, purse-seine vessels would set their nets around whale sharks, in the knowledge that there would likely be tuna schooling around them. The use of whale sharks as living Fish Aggregation Devices (FADs) is now banned by most tuna regional fisheries management organisations (RFMOs), including in the eastern Pacific (Resolution C-19-06, IATTC 2019) where it is prohibited to set a purse-seine net around a school of tuna known to be swimming around a living whale shark.

**Figure 1.**
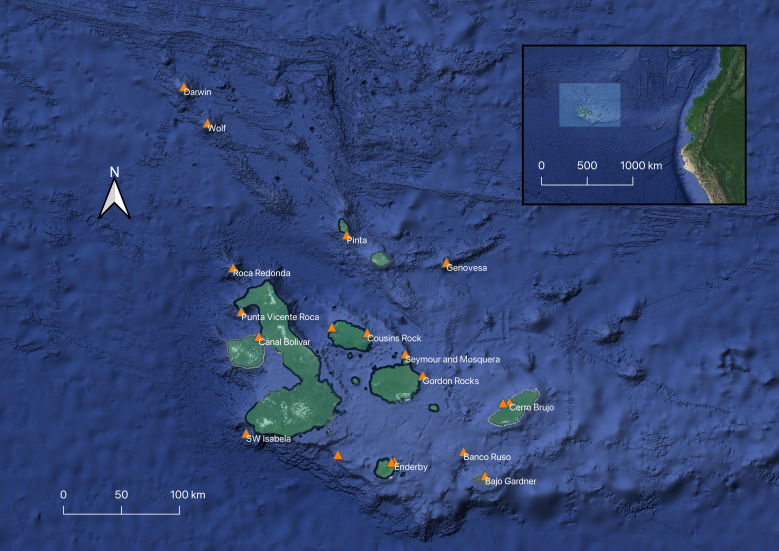
Map of sightings of whale sharks (represented in orange) around the Galapagos Archipelago as reported by dive guides, scientists and fishermen (GWSP-QGIS Development Team 2020).

The eastern Pacific hosts the only known whale shark aggregation made up almost exclusively of large females – the Galapagos Islands (Norman et al. 2017). The first report of a whale shark in the Galapagos was recorded by William Beebe in 1925 of an individual swimming under the stern of a boat on the Arcturus expedition (reported in Gudger (1933)). They continued to be reported occasionally by guides between Floreana and Isabela, in the Canal Bolivar and around another few spots around the Archipelago (see Fig. 1). Prior to whale shark-focused tourism, a short note was written of species associated with whale sharks in the Galapagos Islands by a group of cetacean scientists who spotted four whale sharks by chance during their tracking of sperm whales off the coast of southwest Isabela in 1988. They reported sighting these whale sharks with remoras *Echeneis* spp. around and in the shark's mouth, hammerhead sharks *Sphyrnalewini* (Griffith & Smith, 1834) and tiger sharks *Galeocerdocuvier* (Péron & Lesueur, 1822) swimming close by (at 10 m distance) a whale shark and yellowfin tuna *Thunnusalbacares* (Bonnaterre, 1788) surrounding a whale shark (Arnbom and Papastavrou 1988).

Although seen sporadically throughout the Archipelago, whale sharks occur predictably at the northernmost Island of Darwin, especially in the months of July through November (Acuña-Marrero et al. 2014) and have become an important dive tourism attraction for live-aboard vessels during these months. Since 2011, a team of scientists have led yearly expeditions to Darwin Island to record whale shark movements, behaviour and to attempt to establish their reproductive state. The team also began to collect sightings reports from dive guides from this time. Here, we report on the association of different species with whale sharks at Darwin and across the Galapagos Marine Reserve, in the Eastern Tropical Pacific.

## Methods

### Study Site

The Galapagos Archipelago and its Marine Reserve are a UNESCO Natural World Heritage Site and possess a unique set of characteristics which makes it a hotspot for biodiversity which must be protected. Its isolation from the continent, lying approximately 1000 km due west of mainland Ecuador and its location straddling the Equator, allows for species diversification, which has led to its unique wildlife, with 18% of marine species being endemic (Bustamante et al. 2000). The marine currents that influence the Islands are also important for the diversity of life on the Islands. The Panama current brings warmer waters and carries species from the north, the Humboldt Current brings colder waters and species from the Antarctic and sub-polar region and the Equatorial Under-Current, (also known as the Cromwell Current) brings cold, nutrient-rich water, which upwells along the western margins of the Archipelago. This upwelling generates one of the most productive marine ecosystems in the world (Schaeffer et al. 2008, Smith 2012) which, in turn, attracts highly migratory species, such as the whale shark.

Darwin and Wolf Islands are in the far northern region of the Archipelago and predictable seasonal sightings of whale sharks occur between July and December of every year (Acuña-Marrero et al. 2014).

### Data Collection

For the past decade (2011-2021), the Galapagos Whale Shark Project has led yearly expeditions to this area and collected data on the physiology and ecology of whale sharks and has noted species interactions used for this study. The data collected by the project team come from performing three dives a day every day for two weeks every year.

Data from dive guides and fishermen were collected informally throughout the years. All dive live-aboards in the Galapagos have both Darwin and Wolf in their itineraries where dive guides spend 1-2 days, with 3-4 dives per day, every week in these waters. The dive guides send sporadic informal reports on some notable encounters they have with whale sharks and other species. Fishermen data on the other hand come mostly from the southern and central islands of the Reserve where most of their fishing activities occur. The data collected from their sightings come from word of mouth accounts.

All data presented in this report were collected between 2010 and 2020, except the associations recorded by Arnbom and Papastavrou (1988), which we also include in this note.

During this study, there is mention of species interactions and species associations. For the purposes of this report, we defined associations as an encounter of a species other than *Rhincodontypus*, occurring within 10 m of a whale shark, moving in the same direction and frequently reported being sighted together or physically interacting with the shark. Meanwhile, in ecology, species interactions have been defined as the relationship between two or more species that form an interdependence to some degree. The five major types of species interactions have been labelled as predation, competition, mutualism, commensalism and amensalism (Hillis et al. 2011). Thus, species interactions would be classified as a species association, yet not all associations would be defined as species interactions.

## Results

Nine species, including four other shark species, were found in association with whale sharks in the Galapagos Islands (Table 1) as reported by the GWSP team, dive guides and fishermen of the Reserve.

**Figure 2.**
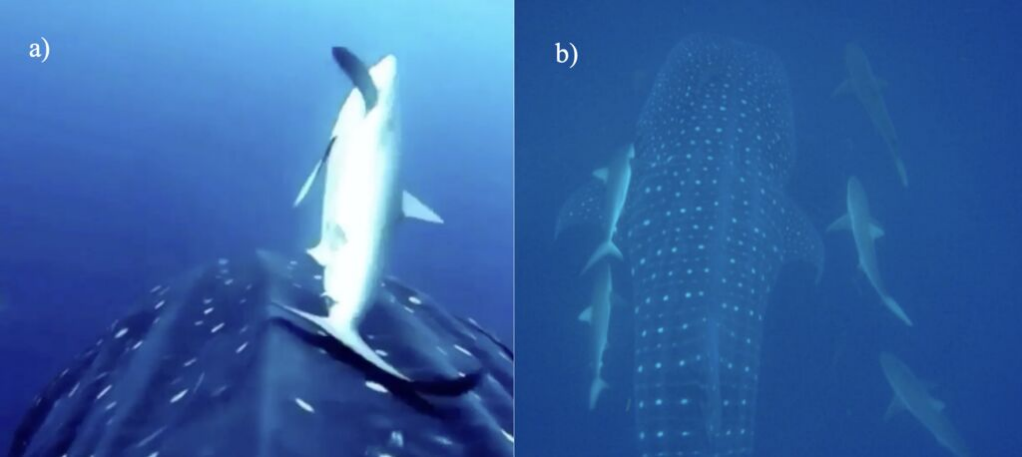
Association of requiem sharks with whale sharks: a) Image capture from BBC bodycam of a silky shark *C.falciformis* rubbing on a whale shark *R.typus*; b) Image capture of Galapagos sharks *C.galapagensis* rubbing on a whale shark and swimming beside it (Photo credit GWSP).

Whale sharks are frequently sighted with other shark species swimming alongside such as *Sphyrnalewini* and the aforementioned *Carcharhinid* sharks. However, *C.galapagensis* and *C.falciformis* have both displayed unusual behaviour around whale sharks not reported elsewhere. Both *Carcharhinus* sp. have been sighted rubbing themselves on the whale sharks’ body and heads on multiple occasions and reported by several Galapagos National Park Guides and the GWSP scientists. This is supported with photographic and video evidence as seen in Figure 2. Both shark species are hypothesised to do this for cleaning purposes, to rid themselves of ectoparasites. Recently, *C.galapagensis*, particularly females, have also been sighted trailing close behind whale shark tails in large groups (4+ individuals, refer to Fig. 2) and displaying agonistic behaviour towards divers when approaching the whale shark (Sofía M Green, pers. observation).

Associations between dolphins and whale sharks have been reported previously by Latusek-Nabholz (2014), off the Gulf of Mexico and have been noted again in the Galapagos Archipelago. Bottlenose dolphins *Tursiopstruncatus* are sighted almost every day at Darwin Island, leading scientists to believe there may be a resident population (data deficient). These dolphins are frequently sighted during dives at Darwin Arch.

**Figure 3.**
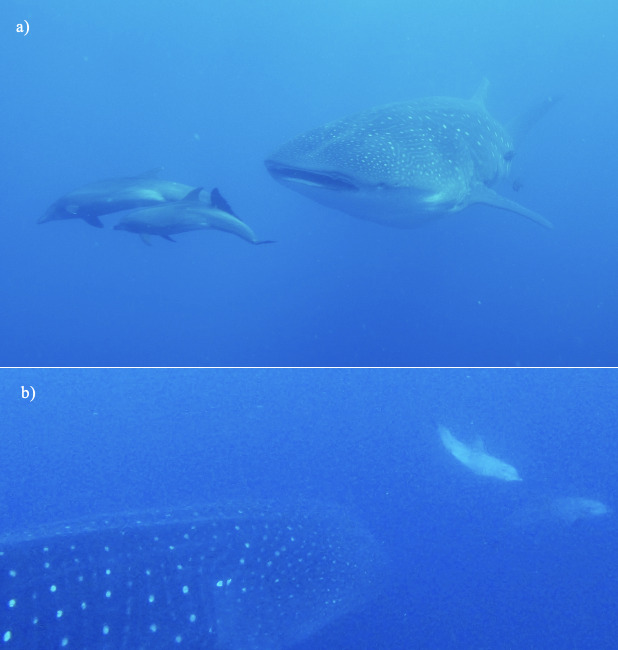
Bottlenose dolphins *Tursiopstruncatus* bow riding whale shark's head: a) 2018; b) 2020 (Photo credit GWSP).

The GWSP has recorded on a number of occasions, particularly in September 2018 and August 2020, dolphins swimming alongside the whale shark and seemingly bow riding its head (Fig. 3). Dive guide Paulo Tobar reported the same behaviour during one of his expeditions to Darwin in 2018.

**Figure 4.**
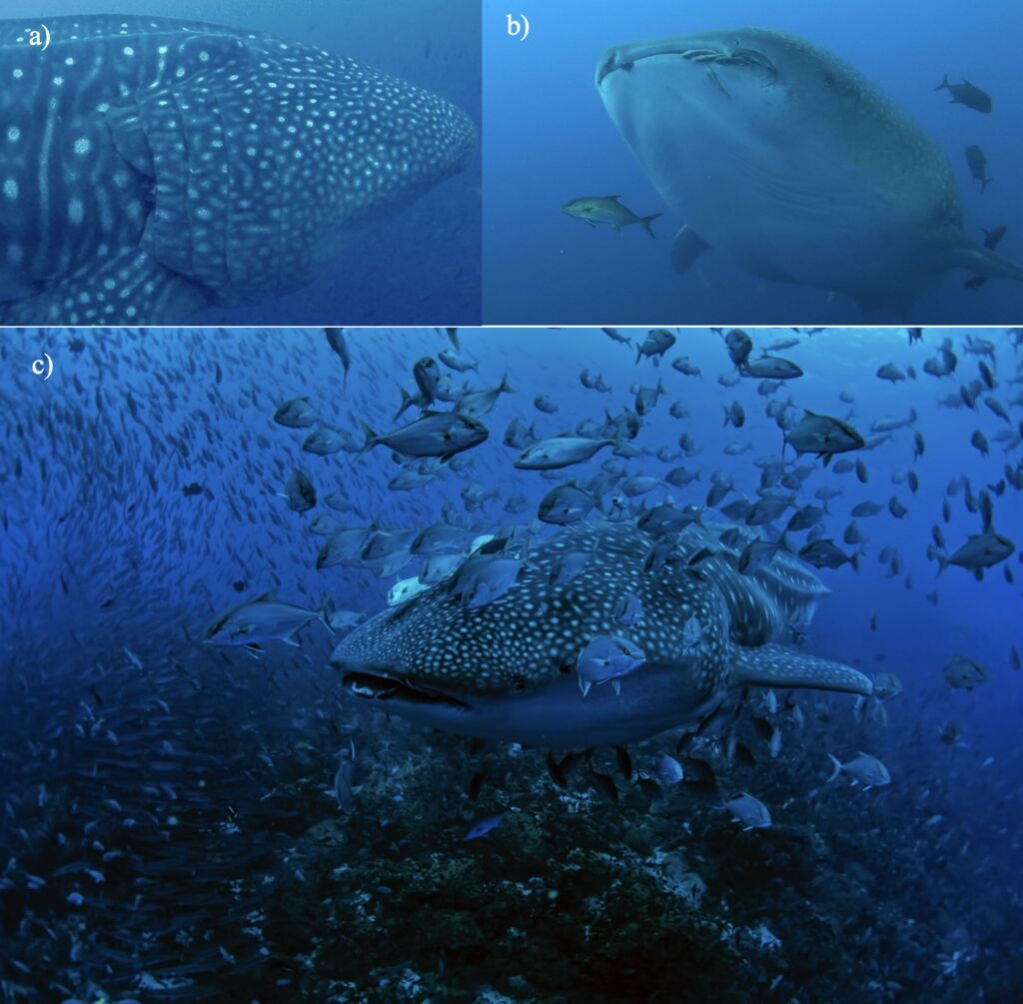
Teleost fish species association: a) Common remoras *Remoraremora* inside the whale shark’s gills; b) Common remoras on mouth with both jack species swimming around; and c) Almaco jacks *Seriolarivoliana* surrounding a whale shark (Photo credit GWSP).

Remora or suckerfish associations have also been reported previously associated with whale sharks around the globe (Colman 1997, Norman 1999, Cohen et al. 2020, Dove and Pierce 2021). This association has previously been noted in the Galapagos Archipelago by Arnbom & Papastavrou in 1988 and again by Galapagos National Park guides and scientists from the GWSP. Common remora’s *Remoraremora* are seen on almost all whale sharks sighted in the islands. They are generally around and inside the whale shark’s mouth, gills and cloaca (see Fig. 4).

Two species of schooling fish have also been sighted frequently in association with the whale shark. In the Galapagos Islands, whale sharks are rarely seen without the company of the Black Jacks *Caranxlugubris* and also frequently sighted with the Almaco Jack *Seriolarivoliana*. These fish are usually surrounding the shark and swimming close to its body (Fig. 4).

## Discussion

Associations between different marine species arise naturally from sharing an environment. In this study, we identified nine species which associate with whale sharks in the Galapagos Marine Reserve. Only one of these appeared to be a long-term association – the remoras affixed to the bodies of the sharks. Two shark species interacted physically with the whale sharks, apparently using their abrasive skin as cleaning tools. The remainder of the associations did not involve physical contact with the shark, but either involved swimming close to the body of the shark (as with the jacks and tunas) or utilising disruptions in water movements caused by the sharks’ movements, as with the dolphins bow riding or the silky sharks swimming in the slipstream. The agonistic display towards the diver by the Galapagos shark may hint to a more meaningful significance for this association with whale sharks. The agonistic behaviour, caused by the rapidly approaching divers was equivalent to the behaviour initially described by Johnson and Nelson (1973) for grey reef sharks and later reinforced by Martin (2007) as noted on other *Carcharhinus* Blainville, 1816 species.

As mentioned by and reported in this study, the sucker fish species not only attach to the whale shark, but live in their mouth, gill slits, cloaca, peribranchial cavity and the spiracle. Although there has been some discussion of whether this symbiosis is mutualistic, parasitic or commensalistic, we report it as parasitic, defined as the relationship where one individual benefits at the expense of the other organism, from sightings observed in the Galápagos. The suckerfish have been observed removing parts from inside the gills, covering the entire mouth and entering the reproductive areas of the sharks, most likely causing damage to the whale shark.

Although around the globe, in Australia, Japan, Indonesia and the Philippines, whale sharks present associations with several other external parastite species, such as copepods and isopods (Norman et al. 2000, Coetzee et al. 2008, Dove and Pierce 2021) as has been demonstrated in the literature collation by Dove and Pierce (2021), no external parasites have been recorded from visual analysis in the Galapagos Islands.

The behaviour of the bottlenose dolphins in the Galapagos, where they are sighted bow riding the shark, is similar to the behaviour reported in the Gulf of Mexico, Belize, Honduras, the Red Sea and Christmas Island, where the mammals were sighted swimming alongside the whale shark (Latusek-Nabholz 2014).

The interactions in each sighting location is based on which species exist naturally in the area and the habitat use of the whale shark. For instance, earlier we mentioned the cleaning behaviour of reef fish, the blue-streak cleaner wrasse *Labroidesdimidiatus* and the moon wrasse *Thalassomalunare* recorded in the Philippines and of the King Angelfish *Holacanthuspasser* in Malpelo Island (Araujo et al. 2020). In Galapagos, although both these species are present, we have not sighted the same behaviour from the cleaner fish with whale sharks, as we do with hammerhead sharks. We suggest this would most likely be due to the fact that the adult whale sharks in the Galapagos Archipelago are sighted in the open ocean and do not spend a significant amount of time on or close to the reefs, while the juvenile whale sharks sighted in the Philippines would. Meanwhile, as mentioned by Araujo et al. (2020), the single sighting of cleaning behaviour in Malpelo Island by the King Angelfish, where whale sharks are also known to be transient and pelagic, is rare and probably opportunistic.

## Figures and Tables

**Table 1.** Species associated with *R.typus* reported in the Galapagos Marine Reserve.

#	**Species Associated**		**Report year**	**Reported by**	# **Reports**	**Behaviour**
	**Common Name**	**Scientific name**				
1	Silky sharks	*Carcharhinusfalciformis* (Bibron, 1839)	2016-2020	GWSP team, Galapagos dive guides	> 10	Sighted frequently rubbing on whale shark’s body and head.
2	Galapagos sharks	*Carcharhinusgalapagensis* (Snodgrass & Heller, 1905)	2019, 2020	GWSP team, Dive guide: Paulo Tobar	> 20	Observed following closely behind sharks. Occasionally also sighted rubbing on the body and head of the whale shark.
3	Hammerhead sharks	*Sphyrnalewini* (Griffith & Smith, 1834)	1988	Arnbom and Papastavrou (1988), GWSP team, dive guide: Paulo Tobar	100+	Observed swimming within 10 m of whale shark and frquently reported being sighted together.
4	Tiger shark	*Galeocerdocuvier* (Péron & Lesueur, 1822)	1988	Arnbom and Papastavrou (1988)	1	Observed swimming within 10 m of whale shark.
5	Bottlenose dolphins	*Tursiopstruncatus* (Montagu, 1821)	2018- 2020	Dive guide: Paulo Tobar, GWSP team	3	Sighted bow-riding whale sharks twice in 2018 and once in 2020. Frequently observed swimming around the shark.
6	Common Remora	*Remoraremora* (Linnaeus, 1758)	1988, 2010-2020	Arnbom and Papastavrou (1988), GWSP team, Dive guide: Natalia Cifuentes	> 100	Attached to whale sharks’ body. Seen mostly around and inside the mouth, caudal fin, pelvic fins of whale sharks. Additionally, spotted inside the gill slits and the cloaca of adult females. Known to do so as an efficient form of transportation and to feed off of left-over food particles.
7	Black jacks	*Caranxlugubris* Poey, 1860	2010-2020	GWSP team	> 100	Swim alongside *R.typus*, possibly making use of slipstream.
8	Almaco jacks	*Seriolarivoliana* Valenciennes, 1833	2020	GWSP team and fishermen	> 50	Swim alongside *R.typus*, possibly making use of slipstream.
9	Yellowfin tuna	*Thunnusalbacares* (Bonnaterre, 1788)	1988	Arnbom and Papastavrou (1988)	1	Surrounding *R.typus*.
